# Beyond symbolic participation: youth-led organisations’ voices and actions against antimicrobial resistance in Africa South of the Sahara

**DOI:** 10.1080/16549716.2025.2601409

**Published:** 2025-12-22

**Authors:** Erick Venant Samwel, Roberta Biasiotto, Michael Mosha, Mirko Ancillotti

**Affiliations:** a Roll Back Antimicrobial Resistance Initiative, Dodoma, Tanzania; b School of Medical Sciences, Faculty of Biology, Medicine and Health, University of Manchester, Manchester, UK; c Department of Biomedical, Metabolic and Neural Sciences, University of Modena and Reggio Emilia, Modena, Italy; d Institute for Biomedicine, Eurac Research, Bolzano, Italy; e Centre for Research Ethics and Bioethics, Department of Public Health and Caring Sciences, Uppsala University, Uppsala, Sweden

**Keywords:** antimicrobial resistance, youth engagement, sub-Saharan Africa, policy, qualitative research

## Abstract

**Background:**

Antimicrobial resistance (AMR) is a global health threat, disproportionately affecting Africa South of the Sahara, where young people have been identified as key stakeholders in AMR prevention and awareness; however, effective engagement beyond symbolic participation remains challenging, limiting the impact of youth-led initiatives in addressing AMR in the region.

**Objectives:**

To examine how youth-led actors in Africa South of the Sahara engage in AMR awareness, education and advocacy; the challenges they encounter; and the institutional conditions needed to move beyond symbolic participation.

**Methods:**

A qualitative study design was employed, involving semi-structured interviews with young professionals engaged in AMR efforts across Africa South of the Sahara. Participants were recruited through convenience and snowball sampling. Online interviews were conducted between March and September 2023. Data were analysed using reflexive thematic analysis.

**Results:**

Seventeen participants (age range 22–34) from 15 countries shared insights on AMR awareness, community engagement, and barriers in their work. Major findings included the perception of AMR as an invisible threat, generational and structural challenges in community outreach, and the need for financial and institutional support. Despite these obstacles, participants demonstrated innovative approaches, including adapting AMR outreach methods to specific social groups and a strong commitment to AMR education and policy advocacy.

**Conclusions:**

Findings underscore the essential role of youth in AMR efforts in Africa South of the Sahara and the need for greater institutional support and capacity-building. Enhancing youth involvement beyond symbolic roles is crucial to advancing AMR initiatives, particularly through tailored communication strategies and collaborative policymaking.

## Paper Context

- **Main findings:** Youth-led organisations across 15 countries in Africa South of Sahara actively support antimicrobial resistance awareness and advocacy but face structural and financial barriers that limit sustained impact.
- **Added knowledge:** The study shows how young professionals tailor communication to different audiences and identifies conditions such as mentorship, funding, and formal roles in decision-making that are needed to move beyond symbolic participation.
- **Global health impact for policy and action:** Recognising and resourcing youth-led work within national action plans, and involving young professionals in policy processes, can strengthen community engagement and more effective antimicrobial resistance responses in low-resource settings.

## Background

Antimicrobial resistance (AMR) poses a significant threat to global health, with a disproportionately high burden in low- and middle-income countries (LMICs). Africa South of the Sahara (i.e. the region commonly referred to in global health and AMR literature as sub-Saharan Africa) has the highest mortality rate worldwide attributable to and associated with bacterial AMR, with an estimated 99 deaths per 100,000 people [1]. The concomitant burden of the ‘big three’ infectious diseases in Africa South of the Sahara, i.e. malaria, HIV, and tuberculosis, along with limited access to effective antibiotics, exacerbates the region’s AMR challenges [2]. The sixty-eighth World Health Assembly approved the Global Action Plan (GAP) and recommended that member states create and implement their National Action Plans (NAPs) to address AMR [3]. However, African nations South of the Sahara meet serious surveillance challenges. These include the absence or lack of implementation of NAPs [4], restricted institutional and infrastructure capacity, low investment and staffing, and inadequate communication with regulatory bodies [5,6]. Additionally, insufficient data and suboptimal data reliability and validity due to a lack of methodological rigour in surveillance systems are considered major challenges to tackling AMR because accurate measurements are needed to implement effective stewardship [7,8].

Translating the objectives of the GAP into meaningful national changes requires the involvement of multiple stakeholders, including youth. The recently adopted UN Political Declaration of the High-Level Meeting on AMR underscores the need for a whole-of-society approach, advocating the engagement of diverse groups, including young people, in the design, implementation, and review of NAPs [9]. Youth represent a vital resource in driving citizen-led initiatives to address AMR, both directly and indirectly [4,10]. Noteworthily, Africa South of the Sahara has the youngest population in the world, with 70% of its inhabitants under the age of 30 [11]. Several programmes exist for youth inclusion in decision-making bodies, such as the UN’s Global Youth Advisory Panel and the Global Fund to Fight AIDS, Tuberculosis, and Malaria. However, to unleash young people’s potential, it is necessary to go beyond symbolic membership and flagship roles [12]. In the AMR field, a remarkable step in this direction was the creation in 2023 of the Working Group on Youth Engagement for Antimicrobial Resistance by the Quadripartite (FAO, UNEP, WHO, and WOAH).

To maximise the impact of youth involvement, it is crucial to understand youth experiences in AMR-related activities. This study aims to examine how youth-led actors in sub-Saharan Africa engage in AMR awareness, education, and advocacy; the challenges they encounter; and the institutional conditions needed to move beyond symbolic participation.

## Methods

### Study design

This qualitative study was grounded in an experiential and contextualist orientation, appropriate for exploring how young professionals understand and describe their engagement with AMR in their local environments. This approach views participants’ accounts as situated within their social and professional contexts. Semi-structured interviews were chosen to allow participants to describe their AMR-related experiences in their own terms and to enable context-sensitive follow-up [13]. Reflexive thematic analysis supported an interpretive reading of the interviews and enabled the identification of recurring patterns in how young professionals made sense of their AMR-related work. Rather than seeking statistical representativeness, the study aimed to generate a contextually informed understanding of youth-led AMR engagement.

This study complies with the consolidated criteria for reporting qualitative studies (COREQ) [14].

### Recruitment of participants

One author (EVS) managed and conducted the recruitment. Being actively involved in the AMR field in Africa South of the Sahara, their extensive network was leveraged to recruit participants through convenience and snowball sampling [15]. Recruitment proceeded iteratively, with additional potential participants identified through the networks of early informants. The inclusion criteria for participating were young age (the African Youth Charter defines youth or young people as people between the ages of 15 and 35) [16], legal age, originating from and residing in the region, possessing relevant professional training or equivalent experience, and having been engaged in AMR work in the region for at least 2 years. Gender balance and geographic variation were pursued. Participants were initially contacted through email, and those who expressed interest were provided with a research information sheet. After addressing any questions, interviews were scheduled. Of the 30 potential participants approached, 13 were excluded for either not meeting the inclusion criteria or not responding within the specified timeframe.

### Interview guide and data collection

A semi-structured interview guide with open-ended questions was developed, which allowed the collection of rich narrative materials using a flexible research design [17]. The interviews focused on participants’ experiences addressing AMR, their perceptions and motivations for implementing AMR programmes, the challenges encountered, and the opportunities envisioned for addressing AMR (see Table 1).

**Table 1.** Interview guide.

**Section 1. Introduction and background**
Please begin by telling me about your experience with AMR programmes.
When did you start engaging in implementing AMR programmes?
What drove you and keeps driving you to engage in implementing AMR-related programmes?
**Section 2. Roles, perceptions, and motivations**
What do you think are the roles of youth and young people in addressing AMR?
What is your opinion about young professionals’ engagement in developing, implementing, and evaluating national action plans on AMR?
How do you feel about collaboration with government stakeholders, private stakeholders, and senior AMR experts?
**Section 3. Experiences in addressing AMR**
Based on your experience in addressing AMR, what has been most meaningful and least meaningful to you (best and worst experiences)?
Do you have experience implementing programmes involving adults and young people, and if so, what are the main differences you noted in their participation or reaction?
As a young professional, can you walk me through how you work with and engage with policymakers on AMR?
Looking back on your experience in implementing AMR programmes, what is needed to improve the engagement of youth at large in addressing AMR?
**Section 4. Challenges and opportunities**
What do you believe are the main issues that need to be addressed to contain AMR?
Are there any specific local issues with the drivers of AMR?
What are some of the challenges you have been facing in addressing AMR programmes?
Are these challenges specific to young professionals? Could you give some examples of what you are saying?
What are the opportunities for addressing AMR in your local context, and what are the gaps?
**Section 5. Closing and additional thoughts**
Any additional thoughts you would like to share?

The interviews were conducted online through Microsoft Teams within the Microsoft 365 platform between March and September 2023. The mean interview duration was 50 minutes (range 40–72). The interviews were conducted in English, without video, and were audio-recorded. The records were transcribed using Microsoft 365 transcription services and then manually reviewed and proofread against the original audio recordings. The principle of thematic saturation guided the sample size. After about 10 interviews, it became clear that the main themes were beginning to repeat. However, interviews continued with the aim of including perspectives from professionals based in various settings across Africa South of the Sahara. This approach was informed by guidance suggesting that when a study involves participants with diverse backgrounds, including geographic and contextual variations, additional interviews may be needed even after saturation seems to have been reached. Francis et al. describe a model in which interviews are extended beyond an initial set to ensure no new themes emerge, especially when participants differ in relevant ways [18]. Saunders et al. similarly note that sample diversity can affect when saturation is reached, and that conceptual completeness may require going beyond early thematic repetition [19]. In this study, the final sample was shaped both by confidence in thematic saturation and the goal of geographic diversity.

### Data analysis

Transcripts were analysed using reflexive thematic analysis with an experiential and contextualist orientation [20,21]. The analysis was conducted with an inductive approach, using semantic and latent coding. The process included the following steps: immersive reading and familiarisation with pseudonymised data; generation of initial codes; generation of themes; review and definition; production of the report. To enhance analytic rigour and trustworthiness, we implemented investigator triangulation through independent coding by multiple researchers and regular discussions to reflect on preliminary themes and challenge assumptions. Three authors (EVS, RB, and MA) participated in the analysis. First, they independently analysed the same sample of three interviews and discussed and compared their coding and preliminary themes. Afterwards, two analysts (EVS and RB) independently analysed seven other different interviews each, holding regular meetings to discuss their impressions and reflections. Further details are provided in the Supplementary file I. The software used for data management during the analysis included ATLAS.ti (version 8.4.26), OpenCode (version 4.03), and Microsoft Excel 2007. Quotations from the interviews are presented in the Results section, organised by theme, illustrating the analysis process and findings through examples [22].

### Reflexivity statement

The author team comprised individuals with diverse disciplinary backgrounds and positionalities from across the Global South and the Global North. Both Global South authors are public and global health specialists with pharmacy backgrounds. One author (EVS), from a country South of the Sahara, is a male youth AMR leader with extensive field experience and a doctoral candidate. His deep engagement shaped the study conception, topic selection, and interview design, drawing on lived experience in youth-led initiatives. His proximity to the field also facilitated participant recruitment and fostered a strong rapport during interviews [23]. While this positionality may introduce bias [23], balanced collaboration mitigated this risk. Another Africa South of the Sahara-based author (MM) contributed perspectives attentive to policy implementation and economic constraints in the region. Two authors based in the Global North (MA and RB) offered more distanced, academically oriented perspectives. MA, a senior researcher in philosophy and public health, guided the project’s conceptual framing and theoretical grounding. RB, with training in biomedical science, medical anthropology, and African studies, contributed insights into communication patterns and social perceptions of AMR. Team reflexivity and triangulation were employed throughout the analysis to strike a balance between insider experience and critical distance. This collaborative process allowed for the emergence of both experiential insights and interpretations shaped through iterative engagement with the data.

## Results

### Respondents’ characteristics

**Figure 1.**
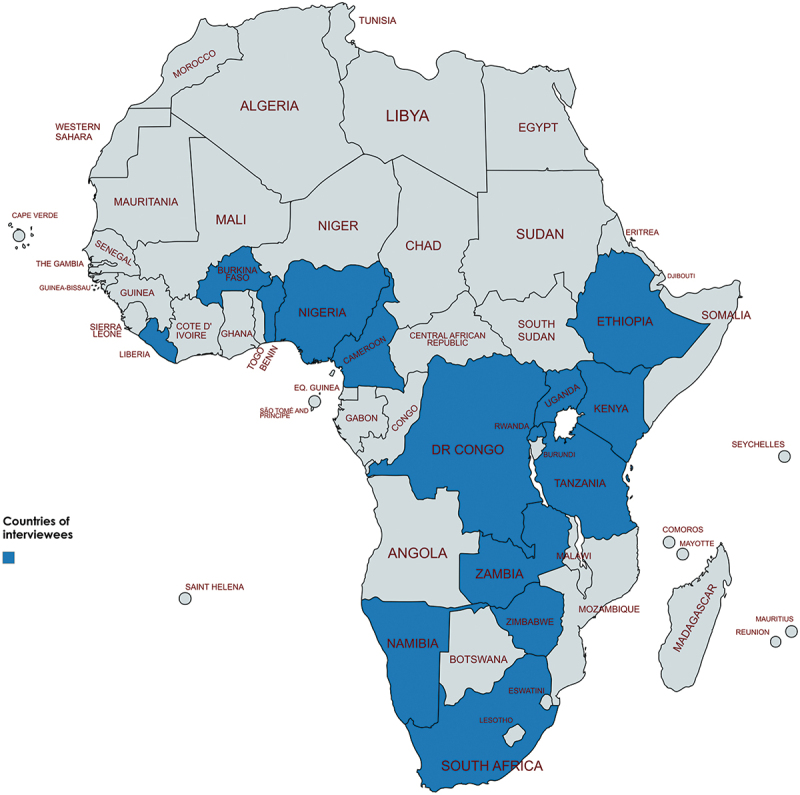
Respondents were based in (in alphabetical order) Benin, Burkina Faso, Cameroon, the Democratic Republic of the Congo (DRC), Ethiopia, Kenya, Liberia, Namibia, Nigeria, Rwanda, South Africa, Tanzania, Uganda, Zambia, and Zimbabwe. All these countries have developed their NAPs. Map created using MapChart.net (https://www.mapchart.net).

Seventeen participants (10 men and 7 women) from 15 countries in Africa South of the Sahara were interviewed (Figure 1). Respondents’ mean age was 26 years (min-max range 22–34). Participants’ professional backgrounds included medical doctors (*n* = 4), medical laboratory scientists (*n* = 2), microbiologists (*n* = 1), pharmacists (*n* = 5), and healthcare students (*n* = 5). Most participants were involved in or led civil society organisations (*n* = 15), while one was in academia and another was a healthcare professional. Participants were actively engaged in various activities to raise awareness and educate diverse audiences (youth, adults, and communities) across multiple contexts, including rural areas and universities.

### Themes

#### The analysis yielded five themes

*AMR perception and its determinants*: Respondents identified AMR as an overlooked issue. They envisioned individual behaviour and structural determinants as factors contributing to AMR.

*Response to and impact of youth-led interventions*: Youth-led interventions were received with different degrees of interest and enthusiasm according to their target (variance in generational response). Respondents described youth-led interventions as impactful and considered strategies for improvement.

*Barriers and challenges of youth-led interventions*: Respondents lamented financial barriers, reluctance, and a lack of support from key stakeholders. They felt that youth were being left out of decision-making roles or included in ways that were tokenistic or instrumental, with limited real influence on decisions or outcomes. They also reflected on the challenges posed by the youth’s attitudes and skills.

*What should be done next – curbing AMR as a collective effort*: Curbing AMR was conceived as a collective effort requiring a comprehensive strategy, addressing structural determinants and increasing education.

*The role of youth*: Respondents envisioned youth as innovators and agents of change. To fulfil this role effectively, they emphasised the need for greater engagement and for youth to take on more comprehensive responsibilities.

Tables 2–6 contain quotations from the interviews organised by theme. Quotation numbering (Qn) follows the order of appearance in this text. Interviewee numbering (In) follows the temporal order of interview completion.

**Table 2.** Exemplar quotations of the theme ‘AMR perception and its determinants’.

Number	Excerpt
Q1(I8)	*It seems more influenced by the structure of how we perceive diseases. HIV and malaria have identifiable faces and symptoms that people can relate to. For instance, a febrile child is a common image associated with malaria. HIV, with its historical impact, also carries a recognisable image. … In contrast, AMR, with its complex microbiological aspects, lacks a straightforward face. It becomes challenging for people to engage with it personally, as it requires a deeper understanding. So, unintentionally, other diseases take precedence due to their visible and relatable nature.*
Q2(I10)	*People don’t do testing, but you put it in our context here … It would be a viral infection. It would be a fungal infection or some parasitic infection. But then, everyone would take antibiotics. Because they feel that it’s antibiotics, they will do the work. So much so that people would take antibiotics for a cold or anything.*
Q3(I14)	*While policies have been drafted, they have not been effectively integrated into the system, and the main issue of financial strain on those responsible for implementing them remains unaddressed. … Regulations are there, and people are not allowed to purchase antibiotics without a prescription. But they are not being implemented or enforced, and people can purchase antibiotics over the counter.*

**Table 3.** Exemplar quotations of the theme ‘Response to and impact of youth-led initiatives’.

Number	Excerpt
Q4(I17)	*When addressing AMR with young people, using animation videos and online campaigns works well. They are more eager, enthusiastic, and participatory. On the contrary, the older generation prefers face-to-face conversations in the local language. Engaging older individuals can be challenging due to ingrained patterns, making it trickier for them to understand and implement the recommended changes.*
Q5(I8)	*… [T]he most meaningful moments revolve around witnessing individuals grow holistically. When I see young people evolve from being unaware to actively engaging in community projects, winning fellowships, grants, and scholarships, and becoming influential, those are the highlights for me. It’s inspiring to observe their passion and dedication.*
Q6(I11)	*We work with them [policy makers] every time we organise an event; we try to have them. … Because they need to know what is being done … at the regional level and at the global level. To, yeah, to let them know that there is definitely a gap that needs to be bridged … The fact that we have field experience and community experience, as well as legitimate data from those experiences, definitely helps to strengthen our efforts.*

**Table 4.** Exemplar quotations of the theme ‘Barriers and challenges of youth-led initiatives’.

Number	Excerpt
Q7(I16)	*Nevertheless, there is a persistent challenge in the process, mainly attributed to the scarcity of funding for projects. While youth are brimming with creative ideas, the crucial step of turning these ideas into reality often requires financial backing. Regrettably, not all projects receive the necessary funding, and this issue stands as the primary barrier, based on my experience. Certainly, there are other factors that can hinder funding for programs, but in my observations, the lack of financial support remains the most significant obstacle. It prevents many promising youth-led initiatives from advancing beyond the development stage and realising their full potential in terms of both implementation and long-term sustainability.*
Q8(I13)	*I designed a project to promote education and awareness, and I sent the proposal to university management and experts but received no feedback. There’s a lack of mentorship, motivation, and engagement from professionals. … Additionally, young professionals face challenges in getting responses or encouragement for their projects from established experts.*
Q9(I8)	*One major hurdle is the difficulty in getting young people committed to AMR initiatives. The aggressive job market and high unemployment rates push them to engage in multiple activities, making it challenging to focus and commit fully. This lack of commitment affects project completion and the overall experience gained.*

**Table 5.** Exemplar quotations of the theme ‘What should be done next – curbing AMR as a collective effort’.

Number	Excerpt
Q10 (I17)	*My message would be to recognise the urgency of addressing antimicrobial resistance as a collective responsibility. Individuals should prioritise proper hygiene practices, follow prescribed medications judiciously, and spread awareness within their communities. Communities should actively engage in education initiatives and support systemic improvements. Policymakers must prioritise funding for research, infrastructure, and education to create a comprehensive strategy. Remember, preventing AMR starts with individual actions that ripple through communities and nations.*
Q11(I15)	*Infection prevention and control play a crucial role in addressing AMR. Adequate measures are essential to prevent infections, as AMR often develops from such incidents. While there has been progress, especially during the COVID-19 pandemic, there’s still work to be done. Awareness among healthcare professionals and the general public about basic hygiene practices is vital. Strengthening these measures is necessary, and having designated stakeholders or role players in healthcare settings could further enhance infection prevention and control.*

**Table 6.** Exemplar quotations of the theme ‘The role of youth’.

Number	Excerpt
Q12 (I10)	*I believe that to address AMR effectively … it’s essential to involve young people comprehensively. Our population includes a significant percentage of young individuals, and their engagement is crucial. … Many of them are young professionals, recent graduates, or college students, and they can be the driving force behind these efforts. Young people should play a role in creating awareness, conducting testing, surveillance, and even drafting policies related to AMR.*
Q13 (I16)	*Young people have great ideas, but they often need the government’s support to put these ideas into action. Some programs can be funded by private organisations and NGOs, and they’re working on getting that funding. However, it’s essential for the government to encourage and fund young people involved in addressing AMR. This approach can be more cost-effective for the government because young people are better at connecting with local communities, especially through their peers.*

#### AMR perception and its determinants

AMR was conceived within the notion that the perception of a problem affects its prioritisation. Due to its ‘invisibility’, AMR was described as an overlooked health issue (Table 2, Q1). When respondents described AMR, they referred to it in terms of scarcity, i.e. lack of public awareness and discourse on AMR, often overshadowed by other pressing health issues. Also, they perceived a lack of depth in how AMR is considered (e.g. lack of attention to environmental aspects) and a scarcity of education, research, and activities to curb AMR. Foremost, AMR would not be prioritised by governments and politicians, leading to unclear or insufficient implementation of the NAPs.

Respondents indicated both individual behaviour and structural determinants as drivers of AMR. People’s misconceptions about antibiotics fuelled misuse, including self-medication (Table 2, Q2). In the healthcare sector, limited resources, poor coordination, and unaffordability have left it ill-equipped to address AMR. Infection prevention strategies, which are key to controlling AMR, were perceived as inadequate due to the lack of measures and guidelines and to healthcare professionals’ limited knowledge of infection prevention. The lack of oversight of the medicine market and dispensing allowed over-the-counter access to antibiotics (Table 2, Q3).

Many respondents described AMR as overlooked, not because people doubt its seriousness, but because everyday realities in healthcare, public understanding, and policy implementation make it difficult to recognise and address the problem.

#### Response to and impact of youth-led interventions

Respondents described the response, intended as attitudes of the target population, to their awareness initiatives. A generational difference regarding expressed interest, engagement, and attitude towards change characterised societal response. Adults and older adults showed relatively good capacities to understand complex information, but were more difficult to engage in, especially if activities were facilitated by young people, and showed less interest and were less inclined towards behaviour change. Overall, younger people were easier to engage, more interested, and more likely to change their behaviour. Respondents used tailored communication strategies with different media and types of language according to whom they addressed and the context (Table 3, Q4).

Reflecting on the impact of their interventions, respondents found that collaboration with young professionals and the government was fruitful, with both stakeholders participating in and engaging in youth-led interventions. Overall, they appraised the interventions as successful, resulting in increased awareness and knowledge and flourishing engagement (Table 3, Q5). Respondents were attentive to strategies to amplify their impact, such as transgenerational (e.g. transfer of information from youth exposed to AMR awareness interventions to adults within the same household) or trans-contextual (e.g. from university students to communities) concatenation of awareness. Respondents advocated for youth involvement in policymaking through their activities and expertise in public engagement (Table 3, Q6).

Across the interviews, it became clear that the impact of youth-led initiatives was shaped not only by the content of the messages but also by how different generations relate to the idea of being guided by young people, including whether they view these young facilitators as credible and worth listening to. This intergenerational dynamic often shaped both engagement and the limits of what such interventions could achieve.

#### Barriers and challenges of youth-led interventions

Respondents described financial constraints as one of the main barriers to youth-led interventions. According to them, the lack of funding affected both the implementation and sustainability of interventions (Table 4, Q7). Other barriers emerged in the cooperation and relationship with different stakeholders. Respondents perceived a lack of interest and reluctance on the part of the government, the Ministry of Health, senior professionals, and academics to collaborate with youth in AMR interventions. They also perceived a lack of support from these expert and experienced stakeholders (Table 4, Q8). Youth were not involved or involved in a limited way in decision-making and policy, such as NAPs and technical committees. Respondents believed that the other stakeholders often underestimated youth and young professionals because of their youth status, hindering their potential.

On the other hand, respondents reflected on how youth perceived themselves, highlighting that they felt ill-equipped in communication skills or lacked credibility. They also criticised the young people who lack commitment and willingness to engage (Table 4, Q9).

Interviews repeatedly pointed to a broader imbalance in expectations: youth are encouraged to take initiative in AMR work, yet they do so within systems where resources, mentorship, and formal authority remain scarce, making it difficult for their ideas to translate into lasting influence.

#### What should be done next – curbing AMR as a collective effort

Curbing AMR was perceived as a collective effort and responsibility of individuals, communities, institutions and organisations, government, policymakers, and experts, which required a coordinated and comprehensive strategy through policy, awareness, infrastructure, and research (Table 5, Q10). Respondents made numerous suggestions emphasising that responsible behaviour depends on the extent to which structural and systemic determinants are addressed. Consequently, strengthening infection prevention, improving water and sanitation, and fighting poverty would decrease infections and the need to access drugs in the first place (Table 5, Q11). Making healthcare affordable and overseeing drug dispensation would limit the misuse of drugs. These improvements must be supported by improved education in AMR at all education levels and training for healthcare professionals. Respondents also stressed that more AMR interventions and coordination among existing organisations and initiatives are needed.

While respondents saw a role for individual and community action, they also viewed such efforts as only effective when supported by stronger systems, coordinated policies, and the basic conditions needed for people to act differently.

#### The role of youth

At an ideal level, respondents considered youth as carriers of change and innovation, capable of providing fresh ideas and energy to counter the spread of AMR. Youth should be acknowledged as partners for their expertise and capability. Youth were expected to be responsible and proactive in their engagement in AMR initiatives. Higher youth engagement was perceived as key to broader societal awareness of AMR. According to respondents, youth should be involved more comprehensively, e.g. in research and policymaking (Table 6, Q12). They reflected that youth involvement in activities should go beyond simply assigning tasks or using youth as tools to achieve goals. Instead, there should be a focus on actively engaging and empowering youth, ensuring they have a genuine stake in the activities and are equipped with the necessary support and resources to sustain their involvement effectively. Capacity strengthening through tailored training (e.g. project management and communication expertise) and collaborations with government, institutions, and NGOs at the national or international levels were considered crucial for the effectiveness and sustainability of youth-led interventions (i.e. securing financial support and amplifying impact) (Table 6, Q13).

Therefore, youth are portrayed as drivers of change, yet their actual role is shaped by whether institutions and senior actors recognise their expertise, grant them meaningful decision-making space, and provide the conditions for long-term engagement.

## Discussion

Africa South of the Sahara countries face distinctive LMIC challenges in implementing effective and sustainable AMR surveillance programmes. As confirmed by this study, these challenges include inadequate infrastructural and institutional capacities, insufficient investment and human resources, underutilisation of available data, limited dissemination of information to regulatory bodies, poor regulation, and enforcement of laws (including the over-the-counter sale of antibiotics), inadequate infection prevention and control (IPC) measures, insufficient Water, Sanitation, and Hygiene (WASH) infrastructure, weak diagnostic capabilities, and low awareness and understanding of AMR [2,5–8]. Despite extensive evidence of the severe current and future impacts of AMR, in low-resource countries within the region, the execution of NAPs is often inadequate and characterised by deficient actionable measures and weak accountability [24,25]. Although implementing NAPs requires significant structural interventions, some actions include cost-effective strategies, such as promoting hand hygiene and raising public awareness, which can significantly curb AMR with a minimal financial burden [26]. Interviewees emphasised the critical role of infection prevention in mitigating AMR, noting that preventing infections is one of the most effective means of controlling its emergence and spread. However, most countries in Africa South of the Sahara lack fully functional WASH or environmental health standards across all healthcare facilities, and only a few countries in the region have established IPC programmes for animals [6]. Additionally, the participants in this study highlighted that diseases such as malaria and HIV/AIDS overshadow AMR due to their significant burden, thus making AMR not a priority compared to other competing public health challenges, as noted by previous research [6]. This is concerning because these diseases are becoming increasingly difficult to treat due to the emergence and spread of resistant organisms [27].

Just as the containment of COVID-19 required extensive global collaboration and commitment, combating AMR would demand a similar level of international effort. Resistant organisms can spread across borders, making AMR a global threat affecting any country. Governments, particularly in LMICs, must prioritise AMR and increase resource allocations to support the implementation of NAPs and cross-border collaboration. The failure to effectively execute these policies exacerbates the threat posed by AMR. High-income countries can further contribute by enhancing financial support for the AMR Multi-Partner Trust Fund and aiding in implementing NAPs in LMICs.

### Substantive youth engagement in AMR

Participants focused their initiatives on awareness and communication on AMR, intending to increase knowledge and challenge misconceptions about AMR, ultimately impacting individual behaviour. When considering the reception of their initiatives, participants expressed overall satisfaction with the impact and effectiveness of their activities. However, they reported differences in response according to the targeted interlocutors: young people responded with enthusiasm and an open attitude to change, while adults were less likely to engage in activities and less keen to change behaviour if activities were led or facilitated by young professionals. Additionally, participants identified different preferences for communication strategies in these societal groups, with social media and messaging widely used by the younger generation vs face-to-face communication in local languages privileged by the older generation. To increase societal engagement and broaden the audience, youth-led interventions should strengthen the adoption of a structured tailoring approach according to the target, with multimedia strategies, and attentive to the distinct preferences of adults and young people, which will amplify youth-led interventions’ impact on societal awareness and education effectively. This is in line with previous research showing that health messages delivered by communicators who share similar age-related concerns are more effective in influencing behaviour across different age groups [28,29]. Building on participants’ insights about trans-generational and trans-contextual concatenation of awareness, young people in Africa South of the Sahara, especially young women, often act as family information hubs. Their greater proficiency with mobile phones and consistent engagement with messaging platforms make them key figures for communication, fostering engagement, awareness, and behaviour change [30].

When participants described their activities, a network of interactions and relationships emerged, not only with the recipients of their outreach but also with health organisations and decision-makers. Participants expressed concerns that their youth status presented challenges in establishing more productive interactions within the AMR field. Despite efforts to build networks, collaborate with key stakeholders, and leverage their extensive field experience in awareness and communication across diverse contexts – as well as their educational background, leadership skills, and expertise in AMR – they often felt undervalued. Instead of being recognised as essential contributors, they were frequently relegated to the role of implementers. Participants highlighted the reluctance of important stakeholders to allocate adequate resources to support youth engagement, pointing to a concerning pattern where young people’s voices are not given enough weight. This disengagement can trigger a cycle of marginalisation, disempowering youth and diminishing their motivation to contribute, thereby stifling the creative ideas they might offer. The findings resonate with a vast literature showing that even in participatory approaches with youth, children and young people are often little involved and able to influence the development of interventions targeting health and well-being [31,32]. Although fostering youth engagement in research and health promotion has been shown to improve health outcomes, self-perception, and a sense of responsibility within communities, practical approaches to purposeful and sustainable youth involvement remain limited [33]. Our findings highlight that the issue of limited meaningful participation in health-sector initiatives affects not only younger age groups (ages 10–24) often recruited as study participants, as traditionally studied and for whose involvement most participatory models are built [34], but also young professionals. This underscores the need for expanded frameworks that support active roles for early-career professionals in decision-making and intervention development within the AMR field.

In addition, this study raises concerns that some young people may lack the knowledge or self-assurance required to engage in decision-making fully. This perceived inadequacy can further reduce their willingness to participate, perpetuating a self-fulfilling prophecy of disengagement. To overcome this, stakeholders must not only provide financial support but also invest in mentorship and capacity-building, and training programmes would be needed to equip young people with the skills needed to take on decision-making responsibilities.

Despite growing opportunities for youth involvement and decades of emphasis on building youth participatory action approaches [35], significant gaps remain in vision and support for youth-led programmes and organisations against AMR.

### Strengths and limitations

This study pursued analytic rigour as a measure of quality [21]. To strengthen the credibility of the study, investigator triangulation measures were implemented, as described in the Methods. A detailed account of the Methods and Results was provided to support transparency and allow readers to assess the relevance and applicability of the findings. While efforts were made to ensure diversity in the sample through carefully defined inclusion and exclusion criteria, purposive and snowball sampling, as forms of convenience sampling, are sometimes critiqued for their limitations in external validity. Additionally, not all Africa South of the Sahara countries were represented in this study, and the sample was limited to individuals with a background in human health. Future research should include professionals from a wider array of fields, especially given that addressing AMR requires a One Health approach that integrates expertise beyond human health.

## Conclusions

This study shed light on how AMR can be perceived as invisible, manifesting in various ways: while the public may feel disconnected from the issue, health professionals may lack adequate training, and public health decision-makers are often occupied with competing health priorities. Emphasising the threat that drug resistance poses to well-known diseases, such as HIV/AIDS, malaria, typhoid, cancer, and gonorrhoea, can help elevate AMR’s visibility among both the public and policymakers. Additionally, sharing patient stories from communities impacted by drug-resistant infections can bring a personal, tangible dimension to AMR, underscoring its urgency. Addressing AMR is a collective responsibility involving lay people, communities, health professionals, policymakers, and the research sector. Strengthening funding and capacity-building programmes to support youth-led AMR initiatives is essential. As key drivers of social change, youth hold unique potential for advocacy and outreach in AMR awareness efforts. The findings highlight the need to adapt AMR interventions and communication to the diverse preferences and cultural contexts of target audiences, including tailoring communication channels and messengers accordingly. Given their capacity for societal impact and knowledge transfer, youth should be offered substantial roles in policy and decision-making processes. Moving beyond a participatory approach that risks being only symbolic, substantial and meaningful youth engagement will enhance the capabilities of young people, broaden opportunities for their involvement, and create supportive environments to facilitate their contributions to NAP implementations.

## Supplementary Material

Supplementary file.docx

COREQ.docx

## Data Availability

Due to confidentiality and GDPR constraints, full transcripts are not publicly available. De-identified data may be shared on reasonable request for bona fide research purposes, conditional on ethics approval for secondary use and a data transfer agreement with Uppsala University; requests should be directed to the corresponding author.
