# Supplementary material for: Beyond symbolic participation: youth-led organisations’ voices and actions against antimicrobial resistance in Africa South of the Sahara
Source: Glob Health Action. 2025 Dec 22;18(1):2601409. doi: 10.1080/16549716.2025.2601409 (PMC12724253; doi:10.1080/16549716.2025.2601409)
Supplement: Supplementary file.docx [file ZGHA_A_2601409_SM2473.docx]

# Supplementary file

## Application of Braun and Clarke’s six-step process

EVS, RB, and MA began by immersing themselves in the transcripts, analysing the same three interviews independently (randomly selected). The analysts discussed their coding and identified preliminary themes, observing patterns of meaning. This process helped them familiarise themselves with the data and agree on the general direction of the analysis.

Familiarisation with the data

During the initial analysis, unique codes were attributed to excerpts from the text. EVS, RB, and MA agreed on coding strategies. It is worth emphasising that diverse perspectives enhanced the depth of the analysis. This phase included systematic coding of the first three interviews, which provided a foundation for analysing additional data.

Generating initial codes

After completing the initial coding, EVS and RB independently analysed 7 different interviews each, making a total of 14 additional interviews. They held regular discussions to refine their impressions and reflect on emerging patterns. RB drafted the main preliminary themes, synthesising the coding work and considering the observations from EVS.

Searching for themes

Preliminary themes were reviewed and finalised in collaborative discussions involving EVS, RB, and MA. Subthemes were refined, and broader themes were agreed upon through iterative dialogue. This step ensured coherence between the themes and the dataset.

Reviewing themes

The finalised themes and subthemes were clearly defined and contextualised. EVS and RB selected representative quotes to illustrate the identified themes, which were discussed with MA and MM. This process ensured the themes effectively captured the dataset’s core narratives.

Defining and naming themes

EVS, RB, and MA compiled the final report, incorporating the selected representative quotes and finalised themes. This report underwent review by MM, ensuring its alignment with the analysis and thematic framework.

Producing the report
